# Supplementary figures and images for: Remeshing flexible membranes under the control of free energy
Source: PLoS Comput Biol. 2022 Dec 5;18(12):e1010766. doi: 10.1371/journal.pcbi.1010766 (PMC9754615; doi:10.1371/journal.pcbi.1010766)

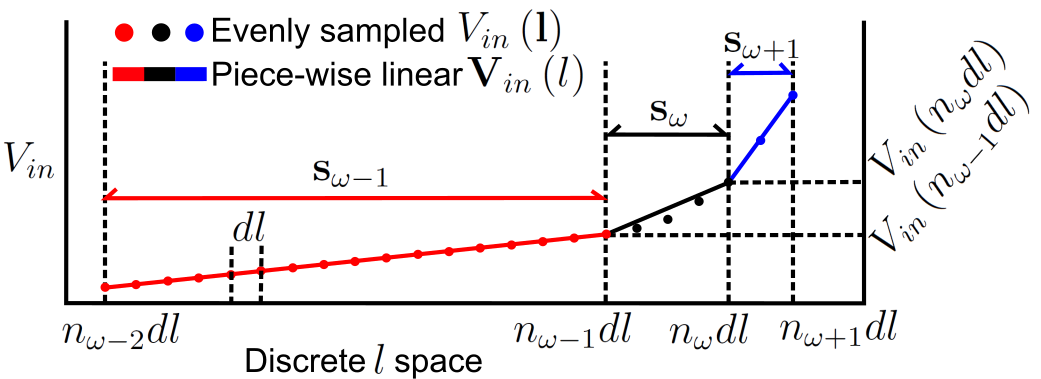

Supplement: S1 Fig — l-space discretization and segmentation according to Vin: examples of complex regions of Vin in black (non-linear) and blue (highly varying) segments and regular regions of Vin in red segments. (TIF) [file pcbi.1010766.s001.tif]

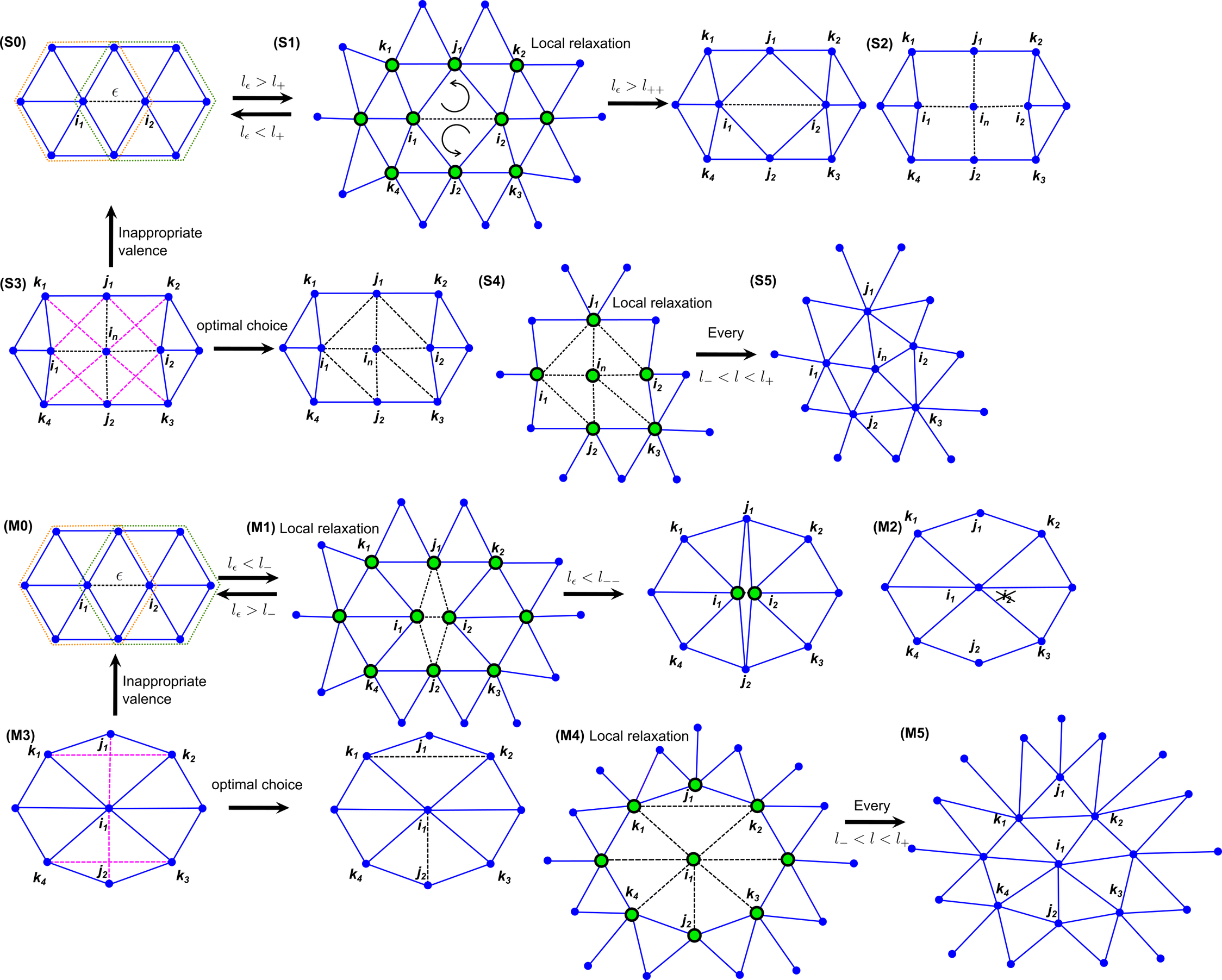

Supplement: S2 Fig — Initial configuration (S0) and steps of splitting (S1-S5); and initial configuration (M0) and steps of merging (M1-M5). See text for detailed procedures. (TIF) [file pcbi.1010766.s002.tif]

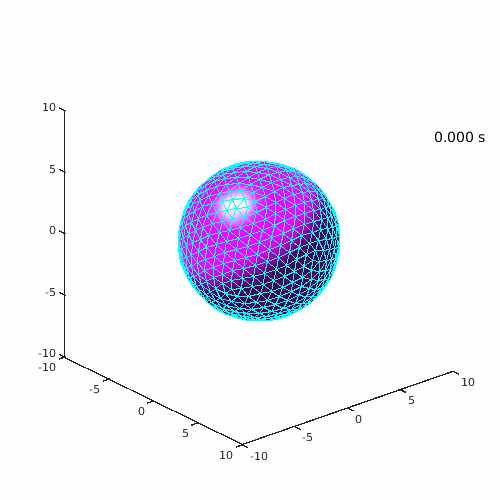

Supplement: S1 Video — Video matching Fig 3A. (GIF) [file pcbi.1010766.s003.gif]

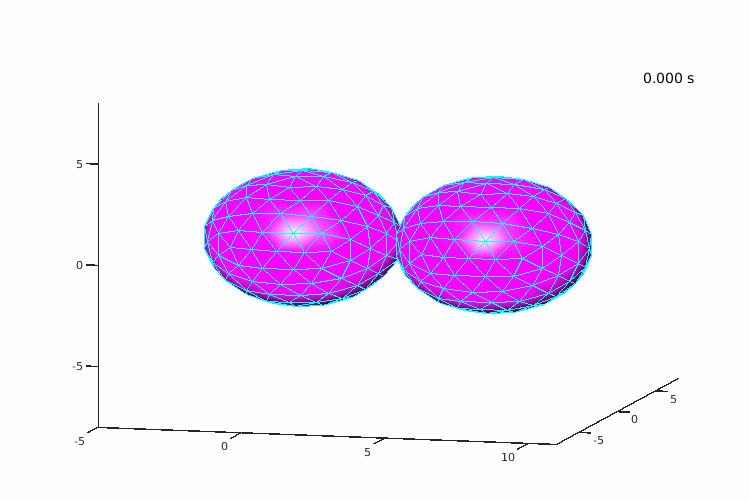

Supplement: S2 Video — Video matching Fig 3B. (GIF) [file pcbi.1010766.s004.gif]

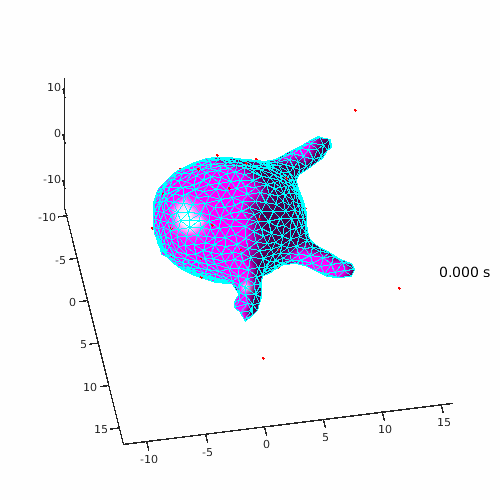

Supplement: S4 Video — Video matching Fig 4B. (GIF) [file pcbi.1010766.s006.gif]

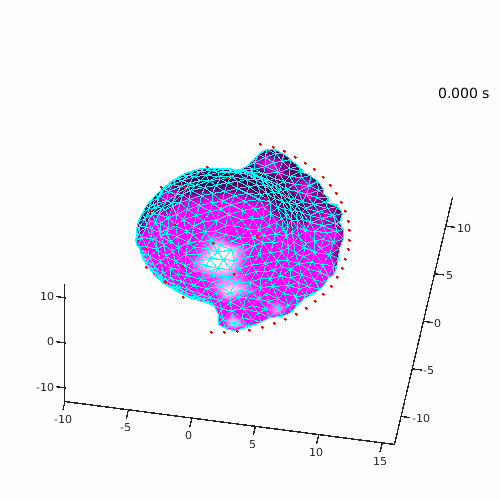

Supplement: S5 Video — Video matching Fig 4C. (GIF) [file pcbi.1010766.s007.gif]

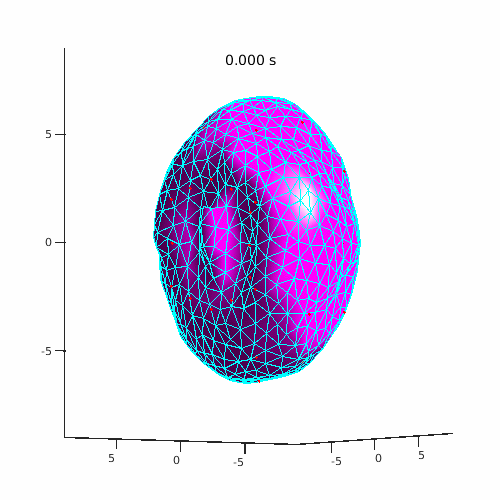

Supplement: S6 Video — Video matching Fig 4D. (GIF) [file pcbi.1010766.s008.gif]
