# Supplementary material for: Remeshing flexible membranes under the control of free energy
Source: PLoS Comput Biol. 2022 Dec 5;18(12):e1010766. doi: 10.1371/journal.pcbi.1010766 (PMC9754615; doi:10.1371/journal.pcbi.1010766)
Supplement: Attachment — Submitted filename: rebuttalLetter.docx [file pcbi.1010766.s009.docx]

Reviewer #1: The following comments are offered to the authors' consideration:

[1.page](http://1.page/) 2, bottom paragraph: The authors wrote that “the computation of the Helfrich free energy is written in C ….”.
Comment: In various Monte-Carlo (MC) and other types of computer programs (including the commercially available computer programs like it is Surface Evolver) the Gaussian bending term or alternatively the deviatoric term (see for example J. Phys. A: Math. Gen., 38: 8527–8536, 2005) cannot be accurately included in the expression of the membrane free energy since it is numerically difficult to determine the Gaussian curvature and also the curvature deviator. The numerical determination of the local mean curvature C (as you have it in Eq. 3) is much easier. The Gaussian term in Helfrich energy can be omitted for closed membrane shapes by applying the **Gauss-Bonnet theorem** (usually for spherical topology) and assuming that the membrane is homogeneous (which is in general not true since the membrane constituents are not homogeneously distributed, see for example V. S. Markin: Lateral organization of membranes and cell shapes, Biophys J., 36(1): 1-19, 1981).
The Gaussian bending term was omitted also in the present work, i.e. in the Helfrich bending energy expression the **Gaussian term is not taken into account** (Eq. 3 in the manuscript) even it is important and cannot be neglected for non-homogeneous membranes, membrane patches, etc.
Further, for the mechanical stability of the highly curved tubular structures (like they are for example the membrane tethers considered in the present manuscript) the anisotropic part of the membrane bending energy (i.e. deviatoric energy term) is important. The anisotropic bending term is the function of the curvature deviator (which can expressed according to Euler formula as a function of the mean curvature and the Gaussian curvature). So also the curvature deviator cannot be taken into account within the method/program presented in this manuscript. See for example Bobrovska et al., PLOS ONE, 8 e73941, 2013 or Kralj-Iglic et al., J. Phys. A: Math. Gen. 35: 1533-1549 , 2002; Fournier, Phys. Rev. Lett. 76, 4436, 1996; Kralj-Iglic et al., Eur. Phys. J. B., 10: 5-8, 1999. Please discuss this issue in the **discussion** part of the manuscript and also in **Introduction**.

We now discuss the issue of omitting the Gaussian curvature in the **Discussion**. However, our **Introduction** does not mention the Helfrich energy and other free energy terms. Therefore, we have opted to not raise this also in the **Introduction**, as it will only lead to confusion. We indicate that the model is only suitable for homogenous and closed membranes and provide feasible ideas for simulating more complex situations. In response to the reviewer’s comment, we tried to add the Gaussian curvature term in Eq (3). As expected, in the configuration of discrete mesh, the requirement for 6 triangles per vertex and similar edge lengths among those triangles can often not be met. This results in a numerical instability that prevents accurate representations of the morphodynamics. This is consistent with the numerical instability of current algorithms of Gaussian curvature using triangular meshes, as pointed out by Reviewer 1. We have opted to not show these failed results. However, we addressed this issue in the **Discussion**.

2.Regarding the relation between the spontaneous curvature model and so-called area-difference elasticity (the authors cited ref. 28 which is not original with respect to these two models), both models are connected, especially in the case of small area difference, see for example Proc. Natl. Acad. Sci. USA, 99: 16766–16769, 2002. So, please discuss also this issue.

We clarify the overlap between the bending energy and the ADE model, and also clarify our choice of using the spontaneous curvature model but not the ADE model for simplicity in the **Results**. Eq (7) and (8) are additional terms that reflect two more energy penalties besides bending and are not related directly to the ADE model. We thus keep the reference.

3.In analytical approach (calculus of variation) the Eqs. (7) and (8) would be global constraints (for cell volume and cell membrane area) in the functional, while k_v and k_s would be the global Lagrange multipliers whose values (determined in variation of the functional by solving Euler-Lagrange differential equations and separately taking into account also the constraint) would depend on the values of the target volume (V_o) and the target area (S_o). Within this approach k_v and k_s are not just the constants describing the mechanical properties of the membrane and cell (see for example Ou-Yang Zhong-Can et al.: Geometric Methods in the Elastic Theory of Membranes in Liquid Crystal Phases, World Scientific, Singapore). Please discuss in the manuscript also this issue.

This approach is continuous and not within the scope of our mesh-based discrete method. Using the same energy terms with the two approaches may lead to different results, as Reviewer 1 pointed out. Considering Kv and Ks as Lagrange multipliers may be less physically interpretable and may require more boundary conditions. To avoid such confusions, we opted not to cite these works.

4.In the present manuscript, the membrane was sculpted externally by setting points external to the bulk of the membrane in order to mimic the tether pulling that is crucial for the study of tension propagation. Even though this is a somewhat artificial constraint, it reflects the corresponding force-curvature feedback mechanisms mentioned in the text. Protruding forces in many cellular processes are recruited through curved proteins, or complexes containing the curved proteins, for example due to actin polymerization. Here, a reference to relevant work should be added, for example the article published in Soft Matter 15, 5319-5330, 2019 or some other article. How could the authors implement such reciprocal mechanism, where the curvature-sensing membrane inclusions produce a protruding force, in the presented work? Is an addition of membrane inclusions a feasible addition to the model?

Clearly, the Reviewer thinks ahead here to the next project. One of many applications of the framework proposed in this manuscript will address the interplay between curvature and force generation. Implementing such models, however, is beyond the scope of the presented work. It would obfuscate the intended didactic modularity. This work is about a numerical scheme for optimal mesh representations of dynamic surfaces that enables a broad range of computational biology projects. Nonetheless, in response to the Reviewer’s comment we offer a proposal in the **Discussion** of how to combine curvature sensing/generating protein structures, such as actin networks or BAR-domain protein scaffolds, with the current membrane model. Due to the restriction on membrane heterogeneity, the current model cannot directly receive changes to intrinsic curvature. However, the model can adapt particle-, mesh- or coarse grained- based components to mechanically change the morphology of the membrane. The curvature readout from the membrane can also indicate how to recruit the curvature proteins to appropriate coordinates on the membrane. Namely, we decompose the reciprocal complex into a pure membrane and other mechanical components and then define their interactions instead of mixing everything into one single model.

5. Could the present model be used to simulate asymmetric membranes in which the mechanics of the outer leaflet differ from the inner one (see Hossein and Deserno, Spontaneous curvature, differential stress, and bending modulus of asymmetric lipid membranes, Biophysical Journal, 118:, 624-642, 2022) and if not, how could this be implemented?

The current model only contains one layer. However, in response to the Reviewer’s question we propose an approach to combining two membrane layers to simulate the double-leaflet structure (see the **Discussion**). The idea is to design a free energy term that describes the interaction between the two layers while keeping all the other operations as defined the original single-layer version. This energy should allow lateral sheer motions but penalize orthogonal motions between the two layers. Asymmetric properties could be assigned to the two layers.

6. The re-meshing mechanics is well described and the three-valley potential is a good idea to assure unbiased meshes. However, the authors could stress more clearly the advantages of their approach to other methods. Is not the re-meshing much more computationally demanding? Furthermore, the authors comment that the method is limited by scaling. How could this be overcome?

1) As a major new addition to manuscript, we now present numerical tests of a geometry (Geo)-based method and compare the result to our new method based on the triple-valley free-energy (FE)-based, see the **Results**. We use the vesicle fusion example for the comparison because of its high interpretability. Although both methods can lead to reasonable results, the Geo-based remeshing requires 10 times more operations than the FE-based method. A reduction of operations is highly desirable for this computation, not primarily for speed, but for accuracy. Each of these discrete operations can cause up to ~10% reduction in accuracy of the computed Helfrich energy even at the optimal mesh quality, see figure 1.

In addition, the Geo-based method produces remeshing events all across the membrane. Compared to the FE-based method, the standard deviation of the coordinates of the events is 2x larger. This discrepancy indicates that the FE-based method is more regulated and performs remeshing only at the hotspots of morphological changes. The FE-based method resolves the rest of the unevenness of the triangles through free-energy determined continuous motions. On the other hand, the Geo-based method relies too much on remeshing and causes uninterpretable and irregular events.

2) To enlarge the scale of treatable membranes, we propose to use either traditional adaptive methods that use different sizes of triangles to treat different areas of the membrane; or neural nets that replace the explicit remeshing with fast heuristic rules that permit faster computations. These points are addressed in the **Discussion**.

7. In the discussion of limitations of current approaches the authors mentioned several authors, however the work of a few more groups could be mentioned within the text and their work included in the discussions. As for example: Gompper and Kroll, “Triangulated-surface models of fluctuating membranes, in: Statistical Mechanics of Membranes and Surfaces, (pp.359-426, World Scientific (Editors: D. Nelson, T. Piran, S. Weinberg); Kroll and Gompper, “The conformation of fluid membranes - Monte-Carlo simulations,” Science, 255: 968-971, 1992; Penic et al., “Bending elasticity of vesicle membranes studied by Monte Carlo simulations of vesicle thermal shape fluctuations,” Soft Matter, 11: 5004 - 5009, 2015; etc.

We cite these papers accordingly.

8.The authors gave the estimation of the computer time for parameter sweep running on a 12 cores, 256GB RAM computer. Besides wall clock metrics of the complexity of the simulations, it would be beneficial to mention how many time steps there were required to achieve the equilibrium states and the size of the mesh, i.e. number of vertices in the mesh (maybe a final vertex number where the number of vertices vary). With the additional data it will be possible to estimate the computational complexity of a single time step of the simulations.

We have added a new sub-section, ‘*Runtime and memory requirements’*, in the **Discussion**, which specifies the RAM cost and run-time of the examples. We also have added iteration numbers in all figure legends.

9. Please explain how much RAM was actually used by simulations? The authors mention only the amount of RAM available on the machine, but not how much RAM was needed by their program. Again, the information about the number of vertices would be also beneficial, since larger vertices means more RAM requirements.

Answer: See the previous answer.

Reviewer #2: The paper by Wang and Danuser presents a new methodology for meshing triangulated membranes. The new approach combines elements of both free-energy and geometry-dependent methods for remeshing dynamic surfaces in computer simulations of processes involving significant morphological changes. The paper, which is well written, has introduced me to a set of important technical issues that I was not aware of in membrane simulations. I believe that it fits the goals of PLOS Computational Biology, and my overall opinion on the work is positive.

I would like to ask the authors to clarify the following technical and conceptual issues:

1. The authors state that their method "preserves mesh quality" on the one hand (like geometry-based simulations) but do so in a free-energy regulated manner. This is indeed the impression the one gets from the nice examples that they provide throughout the paper, but I was wondering if they can give a specific example where other methods fail and the newly presented one works well. Can the author specify, for instance, which of the processes described in the paper **will not be simulated well** by other existing methods?

See the answer to Reviewer 1’s comment #6. We have used the vesicle fusion example to showcase the advantages of the proposed new method over purely geometry (Geo)-based remeshing algorithms.

2. Please also discuss the computational toll of the proposed simulations. In particular, please discuss whether it is comparable to existing popular schemes?

1) For schemes based on free energy, Metropolis-based algorithms are used exclusively. By the nature of the underlying random searches, these schemes are exponentially slower than our gradient-descent-based scheme.

2) For schemes based on geometry, the computational times are similar to the proposed method, although they require 10x more remeshing events. This is because Geo-based remeshing relies on a single operation instead of an iterative optimization to update the positions of the vertices, however at the price of inaccuracy in the mesh representation. This causes errors in computing derivative parameters such as the Helfrich energy, as discussed in the answer to Reviewer 1’s comment #6. See the **Discussion**.

3. The internal energy in Eq. (1) depends on the number of edges of the triangulated surface. Thus, I would expect that it changes when the surface undergo splitting/merging. It is not clear to me if this energy change, which seems artificial, has influence on the dynamics. Please clarify.

After the remeshing operations, the internal energy is controlled below a certain value via local relaxation. Therefore, the dynamics is not significantly affected by the remeshing. Also, the internal energy is not completely artificial, it reflects the adjustment of the lipids upon mechanical perturbations. We have added this point in the description of the internal potential in the **Results**.

4. Related to the above point: In the "three-valley" energy landscape in fig. 2(B), the minimum of the energy is $V_{in}=3$. Is there a reason why the minimum in not shifted to zero? Do the absolute values have importance or only the energy difference between the valleys and the hills matter?

We have removed the minimal energy in the internal potential by adding a balancing parameter V’, see the modification in the **Results**.

5. I found the presentation of the results of Figure 5 confusing.

(a) If I understand correctly, the rate of lipid diffusion is controlled by the constant $k_d$ in Eq. (11). This equation is "empirically" iterated 50 times after remeshing. Can the authors rationalize this choice? Does it mean that the local densities do not continue to change until the next remeshing event?

1) We choose to iterate Eq (11) for N=50 times to compromise between numerical stability and computational cost. Given N, we tuned Kd accordingly. Using larger Kds, we obtain large fluctuations in the density ***ρ***. This is because each iteration of Eq (11) changes ***ρ*** too much. Using smaller Kds, we obtain instabilities in the morphodynamics. This is because we cannot promptly spread out the drastic local changes to ***ρ*** introduced by the remeshing. These changes disturb the morphodynamics according to Eq (12).

2) Also, we stop changing the density after N iterations in between remeshing events. This is for keeping the spreading of ***ρ*** finite so that we can reproduce the experimentally observed mechanical decoupling. Otherwise, the model becomes equivalent to the traditional one. We address these points in the **Results**.

(b) I did not understand the idea of a "wide gap" in Fig. 5(B). How is it implemented and how does it hamper diffusion between the two tethers?

The barrier hampers the diffusion but the gap permits diffusion. The barrier is composed of edges that forbid diffusion. The gap is implemented by allowing diffusion across a subset of edges in the diffusion barrier. The gap captures the real situation in the cell membrane where diffusion barriers usually are leaky. We have rewritten this portion of the text in the **Results**.

(c) Same for Fig. 5(C). How is this idea implemented? Can the authors expand on why the excess membrane decouples between the two tethers,

Having extra membrane allows the membrane from nearby the tether to replenish the tether under mechanical stress lowering the need for membrane exchange with the other tether. This excess membrane mechanically decouples the two tethers. We have addressed this more clearly in the **Results**.
